# Ultra-Long GnRH Agonist Protocol During IVF/ICSI Improves Pregnancy Outcomes in Women With Adenomyosis: A Retrospective Cohort Study

**DOI:** 10.3389/fendo.2021.609771

**Published:** 2021-05-31

**Authors:** Jie Lan, Yaoqiu Wu, Zexuan Wu, Yingchen Wu, Rong Yang, Ying Liu, Haiyan Lin, Xuedan Jiao, Qingxue Zhang

**Affiliations:** ^1^ Department of Gynecology and Obstetrics, Reproductive Medicine Center, Sun Yat-sen Memorial Hospital, Sun Yat-sen University, Guangzhou, China; ^2^ Reproductive Medicine Center, Peking University Shenzhen Hospital, Peking University, Shenzhen, China

**Keywords:** adenomyosis, ultra-long GnRH agonist protocol, long GnRH agonist protocol, IVF/ICSI, pregnancy outcome

## Abstract

**Objective:**

This study aimed to compare the ultra-long gonadotropin-releasing hormone agonist (GnRH-a) protocol and the long GnRH-a protocol during *in vitro* fertilization (IVF) or intracytoplasmic sperm (ICSI) treatment on fertility outcomes in women with adenomyosis.

**Materials and Methods:**

This study was a retrospective cohort study. From January 2011 to May 2018, a total of 371 fresh IVF/ICSI cycles were included. Among the cycles included, 237 cycles of 212 women underwent the ultra-long GnRH-a protocol, while 134 cycles of 116 women underwent the long GnRH-a protocol. The rates of implantation, clinical pregnancy per embryo transfer, live birth, and early miscarriage were estimated between the compared protocols.

**Results:**

In the study, the early miscarriage rate in women undergoing the ultra-long GnRH-a protocol was significantly lower than those undergoing the long GnRH-a protocol (12.0% *versus* 26.5%, *p* = 0.045), whereas the differences in the rates of biochemical pregnancy, implantation, clinical pregnancy, and live birth in women between the two groups showed no statistical significance. The pregnancy outcomes were also sub-analyzed according to the adenomyotic region (diffuse and focal). As for diffuse adenomyosis, the rates of clinical pregnancy and live birth in women undergoing the ultra-long GnRH-a protocol were significantly higher than those undergoing the long GnRH-a protocol (55.3% *versus* 37.9%, *p =* 0.025; 43.4% *versus* 25.9%, *p =* 0.019, respectively). However, pregnancy outcomes showed no difference between the two protocols in women with focal adenomyosis.

**Conclusions:**

The ultra-long GnRH-a protocol during IVF/ICSI improves pregnancy outcomes in women with adenomyosis, especially in women with diffuse adenomyosis when compared with the long GnRH-a protocol.

## Introduction

Adenomyosis is a common cause of menorrhagia and dysmenorrhea, characterized by a benign invasion of the myometrium by the ectopic endometrium and accompanied by hyperplasia of the surrounding smooth muscle myometrial cells (1–3). Adenomyosis is a well-known cause of infertility (4–7). Two recent meta-analyses of assisted reproductive technology (ART) outcomes demonstrated that adenomyosis is associated with lower implantation rates and higher miscarriage rate, translating into an overall significant reduction in the live birth rate (8, 9).

A variety of evidence suggests that impaired uterine function may lead to infertility or poor obstetrical outcomes in women with adenomyosis. Firstly, adenomyosis, even with small implants, causes accumulation of activated macrophages and natural killer cell population density (10, 11), by producing inflammatory mediators and free oxygen radicals in the endometria (12–14), which is harmful to embryos. Secondly, high levels of nitric oxide in the uterine cavity and abnormal expression of protein molecules have been reported in adenomyosis, which is related to impaired implantation (15–18). In addition, hyper-peristaltic uterine contractions are commonly seen in women with adenomyosis, which is linked to increased miscarriage risk (19–21).

Gonadotropin-releasing hormone agonist (GnRH-a) is widely used in adenomyosis therapy, mainly for pain and menorrhagia reduction (22–24). It is well known that adenomyosis grows and declines in an estrogen-dependent manner and GnRH-a produces a period of estrogen deficiency that may temporarily inactivate the lesion. Some research showed that GnRH-a was able to markedly reduce the infiltration of macrophage and micro-vessel density in the endometria, and significantly induce apoptosis in the lesion of adenomyosis (25). Additionally, a direct anti-proliferative effect of GnRH-a may lead to the regression of the lesion (26). Moreover, Guo et al. found that GnRH-a improved the pregnancy outcome by restoring endometrial receptivity in mice with induced adenomyosis (27). Two retrospective studies suggested that GnRH-a pretreatment before frozen embryo transfer increased clinical pregnancy rate in women with adenomyosis (28, 29). Overall, treatment with GnRH-a could be beneficial for IVF/ICSI outcomes in women with adenomyosis. However, it is unknown whether using GnRH-a for a longer period improves the pregnancy outcomes of fresh embryo transferred in women with adenomyosis.

In light of previous findings, the purpose of this retrospective cohort study was to examine the differences in pregnancy outcomes of fresh embryo transferred in women with adenomyosis between those undergoing the ultra-long GnRH-a protocol or those undergoing the long GnRH-a protocol and to determine the preferred regimen in women with adenomyosis undergoing IVF/ICSI.

## Materials and Methods

### Study Cohort

This was a retrospective cohort study. From January 2011 to May 2018, women diagnosed with adenomyosis who accepted their fresh IVF/ICSI cycles at the reproductive medicine center of Sun Yat-sen Memorial Hospital of Sun Yat-sen University were included. Exclusion criteria were: maternal age ≥42 years; previous surgery for adenomyosis; use of GnRH antagonist for controlled ovarian hyperstimulation (COH); use of short GnRH-a protocol or ultra-short GnRH-a protocol for COH; use of mild stimulation for COH; women on their fourth or subsequent IVF cycle. Moreover, women were also excluded if they had uterine malformation, untreated intrauterine lesions, uterine fibroids, or untreated hydrosalpinx.

### Adenomyosis Status

All subjects underwent a screening baseline transvaginal ultrasound between Days 2 and 6 of their menstrual cycle. The diagnosis of adenomyosis was made according to standard radiological criteria (1): enlarged globular uterine configuration, (2) asymmetrical thickening of uterine walls, (3) poor definition of the junctional zone, (4) heterogeneous myometrial texture, and (5) sub-endometrial myometrial striations and cysts (30, 31). In doubtful cases, a pelvic MRI was performed to confirm or refute the diagnosis of adenomyosis (32, 33). In addition, adenomyosis was classified as focal when solitary foci with adenomyotic characteristics were identified. Otherwise, the disease was considered diffuse (22, 34, 35).

### IVF/ICSI Treatment Protocols

The population was divided into two groups according to the treatment strategy adopted (i.e., the ultra-long GnRH-a protocol or the long GnRH-a protocol). The women undergoing the ultra-long GnRH-a protocol received long-acting GnRH-a (Diphereline, Ipsen, France) with a dose of 3.75mg subcutaneously on a monthly basis for 2–4 months. Twenty-eight days after the last dose of Diphereline was administered, ovarian stimulation was started. Conversely, the women undergoing the long GnRH-a protocol received Diphereline at the 18th–20th day with a single dose of 0.93–1.87mg. Fourteen days after Diphereline was given, ovarian stimulation was started.

When endometrium thickness ≤5mm, serum estradiol ≤50pg/mL, and LH ≤5IU/L were confirmed, around 150–300 IU/day gonadotropin (Gonal-F, Merck Serono, Germany; Lishenbao, Lizhu, China; Puregon, MRK, China) was administered according to women’s age, weight, and ovarian reserve. Gonadotropin doses were adjusted according to ovarian response. If necessary, 75–150 IU/day recombinant LH (Luveris, Merck Serono, Germany) was added. Urinary human chorionic gonadotropin (hCG; Lizhu, China) was administered subcutaneously for triggering when at least two follicles measured ≥18mm or three follicles measured ≥17mm. Around 4000–10,000 IU of hCG was given depending on follicular numbers, peak estradiol level, and body mass index (BMI). Oocyte retrieval guided by vaginal ultrasound was performed 34–36 h later.

Ovarian hyper-stimulation cycle was cancelled if poor or hyper-response of the ovaries occurred. If the number of retrieved oocytes exceeded 20 or if in the presence of symptoms and signs, suggestive for ovarian hyper-stimulation syndrome (OHSS) occurred, the fresh transplant cycle was canceled and embryos were frozen. All embryos were cultured for 3 or 5 days before being transferred or cryopreserved. The number of embryos transferred (ET) depended on embryo quality and women’s age. High-quality embryos were defined as Day 3 embryos with 6–10 cells, even cleavage, and <10% cytoplasmic fragments. The luteal phase was supported by 60mg natural progesterone in oil intramuscular injection daily from the day of oocyte retrieval.

### Pregnancy Outcomes

Biochemical pregnancy was defined as positive if β-hCG levels reached more than 25 IU/L 14 days after ET. Clinical pregnancy was defined as the presence of at least one intrauterine gestational sac on the 6-week gestation ultrasound. Live birth was defined as a delivery that resulted in a live neonate. Biochemical pregnancy rate, clinical pregnancy rate, and live birth rate were calculated by per fresh ET cycle. In our study, early miscarriage was defined as fetal delivery at <12 weeks of gestational age. Late miscarriage was defined as fetal delivery at <24 weeks of gestational age. Extreme preterm delivery was defined as fetal delivery at ≥24 weeks but <32 weeks of gestational age. And preterm delivery was defined as fetal delivery at ≥32 weeks but <37 weeks of gestational age.

### Statistical Analysis

The main outcome of the study was the live birth rate. Secondary outcomes were biochemical pregnancy rate, implantation rate, clinical pregnancy rate, early miscarriage rate, late miscarriage rate, extreme preterm delivery rate, preterm delivery rate, and term delivery rate.

Statistical analysis of the data was performed using SPSS version 25.0 (SPSS, Inc., Chicago, IL). Normality quantitative variables are expressed as means ± standard deviations (SD) and were analyzed using Student’s t-test. Non-normal quantitative variables are expressed as median (interquartile range) and were analyzed using Mann–Whitney U test. Qualitative variables are expressed as frequencies (percentages) and were analyzed using Pearson Chi-Square test or Fisher’s exact test as appropriate. *p* < 0.05 was considered statistically significant.

## Results

### Baseline Characteristics

From January 2011 to May 2018, a total of 371 fresh cycles were enrolled according to the designed criteria. Among the cycles included, 237 cycles of 212 women underwent the ultra-long GnRH-a protocol, while 134 cycles of 116 women underwent the long GnRH-a protocol.

The epidemiological characteristics of the study subjects are outlined in **Table 1**. No difference in women’s age, BMI, duration of infertility, basal FSH and LH and E2 level, AMH level, antral follicle count (2–10 mm, AFC), serum CA125 level before downregulation, type of infertility, previous ovarian surgery, and type of adenomyosis were found between the two compared strategies. Meanwhile, statistical differences between the two comparative strategies were observed for uterine volume and associated endometrioma.

**Table 1:** Baseline characteristics of the participants.

Variable	Ultra-long GnRH-a protocol (n=237)	Long GnRH-a protocol (n=134)	*p* value
Age (y)	33.55 ± 4.12	33.99 ± 4.08	0.318
BMI (kg/m^2^)	21.25 ± 2.62	21.35 ± 2.86	0.730
Duration of infertility (y)	5.18 ± 3.95	4.60 ± 3.28	0.136
Basal FSH (IU/L)	8.47 ± 4.04	8.42 ± 3.74	0.903
Basal LH (IU/L)	4.45 ± 2.43	4.72 ± 2.83	0.344
AMH (ng/ml)	2.37 (1.35-4.17)	1.93 (1.13-4.18)	0.111
AFC	12.54 ± 7.44	12.67 ± 7.15	0.873
Uterine volume (ml)	72.16 (52.94-107.04)	61.14 (42.85-90.70)	0.001
CA125 before downregulation (U/mL)	127.23 ± 152.82	108.93 ± 253.75	0.424
Infertility, n (%)			0.808
Primary	111 (46.8)	61 (45.5)	
Secondary	126 (53.2)	73 (54.5)	
Associated endometrioma, n (%)	47 (19.8)	12 (9.0)	0.006
Previous ovarian surgery, n (%)	57 (24.1)	27 (20.1)	0.388
Type of adenomyosis, n (%)			0.826
diffuse	188 (79.3)	105 (78.4)	
focal	49 (20.7)	29 (21.6)	
Etiology, n (%)			
Male factor	3 (1.3)	6 (4.5)	
Tubal	37 (15.6)	23 (17.2)	
Endometriosis	79 (33.3)	28 (20.9)	
Anovulation	5 (2.1)	3 (2.2)	
Mixed/Unexplained/Other	113 (47.7)	74 (55.2)	

Data are presented as mean ± SD, median (interquartile range), or number (%).

BMI, body mass index; AMH, anti-Müllerian hormone; AFC, antral follicle count (2–10 mm); FSH, follicle-stimulating hormone; LH, luteinizing hormone.

### Parameters During IVF/ICSI

The detailed parameters during IVF/ICSI treatment are shown in **Table 2**. The peak E2 level was lower in women undergoing the ultra-long GnRH-a protocol than those undergoing the long GnRH-a protocol (8639.18 pmol/L *versus* 10039.03 pmol/L, *p =* 0.010). Correspondingly, the number of oocytes retrieved was lower in women undergoing the ultra-long GnRH-a protocol than those undergoing the long GnRH-a protocol (8.31 *versus* 9.93, *p =* 0.004). One hundred ninety cycles undergoing the ultra-long GnRH-a protocol and 79 cycles undergoing the long GnRH-a protocol (72.5% of the studied cycles) performed fresh cycle ET. Relevantly, compared with the long GnRH-a protocol, the ultra-long GnRH-a protocol had a lower rate of cancellation of fresh embryo transferred (19.8% *versus* 41.0%, *p =* 0.000). The details of cycle cancellation are shown in **Supplementary Table 1**. Otherwise, there were no significant differences in other IVF/ICSI stimulation parameters between the two compared strategies (e.g., gonadotropin dose and duration, endometrial thickness, number of embryos available or transferred, the embryo quality, stage at ET, and type of ART). Importantly, there was no sign of severe ovarian hyperstimulation syndrome (OHSS) in any women in our study although the two compared strategies showed similar rates of moderate OHSS.

**Table 2:** Cycle characteristics of the participants.

	Ultra-long GnRH-a protocol (n=237)	Long GnRH-a protocol (n=134)	*p* value
Total dose of gonadotropin (IU)	2629.00 ± 858.77	2630.40 ± 913.95	0.988
Duration of stimulation (d)	11.29 ± 2.81	11.31 ± 2.78	0.95
Endometrial thickness in HCG day (mm)	10.82 ± 2.85	10.95 ± 3.00	0.678
Peak E2 (pmol/L)	8639.18 ± 5180.09	10039.03 ± 6883.23	0.01
No. of oocytes retrieved per cycle	8.31 ± 5.42	9.93 ± 7.04	0.004
No. of embryos available per cycle	3.89 ± 2.87	4.34 ± 3.16	0.17
No. of high-quality embryos per cycle	1.56 ± 1.97	1.61 ± 1.73	0.788
High-quality embryos transferred, n (%)	181/389 (46.5)	73/162 (45.1)	0.753
No. of embryos transferred per cycle	2.05 ± 0.57	2.05 ± 0.55	0.965
Stage at ET, n (%)			0.491
Day 3	176/190 (92.6)	75/79 (94.9)	
Day 5	14/190 (7.4)	4/79 (5.1)	
Type of ART, n (%)			0.850
IVF	191/233 (82.0)	101/127 (79.5)	
ICSI	32/233 (13.7)	20/127 (15.7)	
IVF+ICSI	10/233 (4.3)	6/127 (4.7)	
Moderate OHSS (n, %)	3 (1.3)	2 (1.5)	1.000
Cancellation of fresh ET, n (%)	47 (19.8)	55 (41.0)	0.000

Data are presented as mean ± SD or number (%).

ART, assisted reproductive technology; ET, embryos transferred.

IVF, in vitro fertilization; ICSI, intracytoplasmic sperm injection.

### Pregnancy Outcomes

The clinical outcomes in the two compared strategies are shown in **Table 3**. In the study, the early miscarriage rate in women undergoing the ultra-long GnRH-a protocol was significantly lower than those undergoing the long GnRH-a protocol (12.0% *versus* 26.5%, *p* = 0.045), whereas the differences in the rates of biochemical pregnancy, implantation, clinical pregnancy, and live birth in women between the two groups showed no statistical significance. As for the rate of late miscarriage, extreme preterm birth, preterm birth, and term birth, there was no difference found between the two compared protocols. Notably, during the period in which the study subjects had IVF/ICSI treatment, the IVF/ICSI success rates in our clinic for women with tubal infertility in their fresh transplantation cycle, were an implantation rate of 36.27% and a clinical pregnancy rate of 56.59%.

**Table 3:** Clinical outcomes of the participants.

	Ultra-long GnRH-a protocol (n=237)	Long GnRH-a protocol (n=134)	*p* value
Biochemical pregnancy, n (%)	110/190 (57.9)	39/79 (49.4)	0.200
Implantation, n (%)	141/389 (36.2)	49/162 (30.2)	0.177
Clinical pregnancy, n (%)	100/190 (52.6)	34/79 (43.0)	0.152
Live birth, n (%)	79/190 (41.6)	24/79 (30.4)	0.085
Early miscarriage, n (%)	12/100 (12.0)	9/34 (26.5)	0.045
Late miscarriage, n (%)	8/100 (8.0)	1/34 (2.9)	0.448
Extreme preterm birth, n (%)	6/100 (6.0)	2/34 (5.9)	1.000
Preterm birth, n (%)	33/100 (33.0)	7/34 (20.6)	0.172
Term birth, n (%)	41/100 (41.0)	15/34 (44.1)	0.750

Data are presented as number (%).

A sub-analysis was performed on the two protocols according to the adenomyotic region. Among the women with diffuse adenomyosis, 152 cycles undergoing the ultra-long GnRH-a protocol and 58 cycles undergoing the long GnRH-a protocol received fresh cycles transplantation. As for women with focal adenomyosis, 38 cycles undergoing the ultra-long GnRH-a protocol and 21 cycles undergoing the long GnRH-a protocol received fresh cycles transplantation. The clinical outcomes of subgroup analyses between the two protocols are shown in **Table 4**. It is worth noting that significantly higher rates of clinical pregnancy and live birth were observed in women with diffuse adenomyosis undergoing the ultra-long GnRH-a protocol than those undergoing the long GnRH-a protocol (55.3% *versus* 37.9%, *p =* 0.025; 43.4% *versus* 25.9%, *p =* 0.019, respectively). As for women with focal adenomyosis, there were no differences found in clinical outcomes between the two protocols.

**Table 4:** Subgroup analyses on the clinical outcomes.

	**Ultra-long GnRH-a protocol**	**Long GnRH-a protocol**	***p* value**
**Diffuse adenomyosis**			
Clinical pregnancy, n (%)	84/152 (55.3)	22/58 (37.9)	0.025
Live birth, n (%)	66/152 (43.4)	15/58 (25.9)	0.019
Early miscarriage, n (%)	10/84 (11.9)	6/22 (27.3)	0.145
**Focal adenomyosis**			
Clinical pregnancy, n (%)	16/38 (42.1)	12/21 (57.1)	0.268
Live birth, n (%)	13/38 (34.2)	9/21 (42.9)	0.511
Early miscarriage, n (%)	2/16 (12.5)	3/12 (25.0)	0.624

Data are presented as number (%).

## Discussion

The presence of adenomyosis is known to be associated with lower rates of successful implantation, as well as increased risk of early miscarriage (8, 36, 37). Currently, the lack of published randomized controlled trials comparing different IVF protocols represents a major limit in the management of adenomyosis-associated infertility (38, 39). In the study, we demonstrated that adopting the ultra-long GnRH-a protocol in women with adenomyosis reduces the early miscarriage rate compared with the long GnRH-a protocol. In addition, a significantly higher clinical pregnancy rate and live birth rate were observed in women with diffuse adenomyosis who used the ultra-long GnRH-a protocol, suggesting the relevance of IVF protocol for this selected subgroup of women. In contrast, similar pregnancy outcomes were found between both strategies in the subset of women with focal adenomyosis.

In our cohort of patients, those undergoing the ultra-long GnRH-a protocol had larger uterine volume and higher proportion of associated endometrioma than another. Since clinicians tend to choose the ultra-long GnRH-a protocol for women with more severe adenomyosis, non-homogeneous baseline characteristics between the two compared groups are to be expected. Importantly, we speculated that having larger uterine volume and higher proportion of associated endometriomas weakens part of some beneficial effects of the ultra-long GnRH-a protocol on fertility outcomes in women with adenomyosis. We also supposed that those effects led to no statistically significant rates of biochemical pregnancy, implantation, clinical pregnancy, and live birth in women between the two groups. Furthermore, although several baseline characteristics (larger uterine volume and higher proportion of associated endometriomas) of women with adenomyosis undergoing the ultra-long GnRH-a protocol were worse, the early miscarriage rate in women undergoing the ultra-long GnRH-a protocol was significantly lower than those undergoing the long GnRH-a protocol, which implied that the ultra-long GnRH-a protocol had more advantages than the long GnRH-a protocol in women with adenomyosis.

In the study, the implantation rate was 30.2%, and the clinical pregnancy rate was 43.0% in women with adenomyosis who underwent the long GnRH-a protocol. Compared with the clinical outcomes of women with fallopian tube factor infertility only in the same period, adenomyosis did affect the IVF outcomes negatively, which is consistent with the results of several previous studies (40–42). However, the implantation rate and clinical pregnancy rate reached 36.2% and 52.6%, respectively, in women using the ultra-long GnRH-a protocol in the study. In addition, the number of oocytes retrieved was lower in women undergoing the ultra-long GnRH-a protocol than those undergoing the long GnRH-a protocol. Part of the reason could be explained by the deeper suppression of the pituitary function when using the ultra-long GnRH-a protocol. Thus, it was extremely necessary for women with poor ovarian reserve to avoid or only receive with caution the ultra-long GnRH-a protocol. In our clinical routine, clinicians tended to choose the long GnRH-a protocol or other protocols in COH and whole embryos frozen, and then perform frozen thawed embryo transfer cycles following GnRH-a treatment in women with poor ovarian reserves. Moreover, in the study, women using the ultra-long GnRH-a protocol during IVF treatment had lower rates of cancellation of fresh embryos transferred than those using the long GnRH-a protocol. Because endometrial receptivity improved by longer treatment of GnRH-a, it led to more cases of fresh embryos transferred (27, 43, 44). But notably, women using the ultra-long GnRH-a protocol needed a longer treatment cycle than those using the long GnRH-a protocol.

The effects of GnRH-a treatment during IVF in women with adenomyosis have been evaluated in several studies. Park et al. compared GnRH-a treatment *versus* no treatment before fresh ET (28). Niu et al. compared GnRH-a followed by hormone replacement therapy (HRT) or HRT alone for endometrial preparation in women with adenomyosis undergoing frozen ET (29). The results of the two studies showed that treatment of GnRH-a did benefit pregnancy outcomes. However, the design and purpose of those two studies were different from this research. Our study mainly looked at whether women with adenomyosis ameliorated the clinical outcomes of IVF treatment by using GnRH-a for a longer period. In the recent literature, Xiaoni et al. compared the ultra-long GnRH-a protocol and the long GnRH-a protocol during IVF/ICSI treatment on fertility outcomes in women with adenomyosis (45). The results showed that women with adenomyosis undergoing the ultra-long GnRH-a protocol had better pregnancy outcome than those undergoing the long GnRH-a protocol. However, it is critical to state that the designs of these epidemiological studies lead to results inevitably based on significant biases. The most important limitation was the lack of a hierarchical analysis of the baseline characteristics of the subjects of these studies. A recent meta-analysis had shown that different types of adenomyosis do affect the pregnancy outcome of patients with adenomyosis (46). Our study conducted a stratified statistical analysis of patients with different types of adenomyosis. Our results demonstrated that clinical pregnancy rate and live birth rate in women with diffuse adenomyosis undergoing the ultra-long GnRH-a protocol were higher than those undergoing the long GnRH-a protocol. As for women with focal adenomyosis, there were no differences between the two compared protocols. Therefore, based on these results, clinicians could tend to choose the long GnRH-a protocol in patients with focal adenomyosis, because the long GnRH-a protocol took shorter time and cost less than the ultra-long GnRH-a protocol. Our research provides more accurate and personalized references regarding treatment for patients with different types of adenomyosis during the IVF treatment. However, it is worth noting that the sample size of each group was small after subgroup analyzing, which led to moderate or mild statistical power.

In clinical routines, all unfertile women undergoing IVF treatment should receive a baseline pelvic ultrasound to exclude any pelvic pathology. Based on our results and other research, we suggest that it is vital to exclude adenomyosis when patients have high-risk factors such as dysmenorrhea and menorrhagia. We further propose that it is reasonable to consider using the ultra-long GnRH-a protocol as the preferred IVF treatment in adenomyosis patients with normal ovarian function, especially in patients with diffuse adenomyosis. However, our study is mainly limited by its small sample size and retrospective design, which carries inherent potential for bias. Finally, we hope that our study highlights the need for high-quality prospective multi-central randomized controlled trials to be undertaken to provide superior evidence for the potential benefits of the ultra-long GnRH-a protocol.

## Data Availability Statement

The original contributions presented in the study are included in the article/**Supplementary Material**. Further inquiries can be directed to the corresponding author.

## Ethics Statement

The studies involving human participants were reviewed and approved by Institutional Review Board of Sun Yat-Sen memorial hospital. Written informed consent for participation was not required for this study in accordance with the national legislation and the institutional requirements.

## Author Contributions

QZ and JL designed the study and critically revised the manuscript. JL performed data analysis. JL, YQW, ZW, YCW, RY, YL, HL, and XJ collected data. JL, YQW, and YCW drafted the manuscript. All authors reviewed the manuscript. All authors contributed to the article and approved the submitted version.

## Funding

This study was supported by the National Natural Science Foundation of China (81971332); Natural Science Foundation of Guangdong Province (2020A1515011126); and Sun Yat-Sen University Clinical Research 5010 Program (2016004).

## Conflict of Interest

The authors declare that the research was conducted in the absence of any commercial or financial relationships that could be construed as a potential conflict of interest.
