# Supplementary material for: Ultra-Long GnRH Agonist Protocol During IVF/ICSI Improves Pregnancy Outcomes in Women With Adenomyosis: A Retrospective Cohort Study
Source: Front Endocrinol (Lausanne). 2021 May 31;12:609771. doi: 10.3389/fendo.2021.609771 (PMC8202082; doi:10.3389/fendo.2021.609771)
Supplement: Supplementary file 1 [file Table_1.docx]

Supplementary Material

# Supplementary Tables

| **TABLE S1 \| Details of cycles cancellation.** | | |
| --- | --- | --- |
|  | **Ultra-long GnRH-a protocol (n=237)** | **Long GnRH-a protocol (n=134)** |
| **Reason for cycle cancellation, n (%)** |  |  |
| Poor response | 18(7.6) | 10(7.5) |
| Risk of OHSS | 4(1.7） | 4(3.0) |
| Adenomyosis | 1(0.4) | 12(9.0) |
| Increased progesterone levels | 3(1.3) | 1(0.7) |
| Endometrial hydrops or endometrium thinned | 10(4.2) | 5(3.7) |
| Other | 11(4.6) | 23(17.2) |
| *Note: Data are presented as number (%).* | | |
